# CAREGIVING AND SERVICE USE: CULTURAL INFLUENCES, REGIONAL BARRIERS, AND FAMILY RELATIONS

**DOI:** 10.1093/geroni/igz038.668

**Published:** 2019-11-08

**Authors:** Jyoti Savla, Karen A Roberto, Rosemary Blieszner

**Affiliations:** Virginia Tech, Blacksburg, Virginia, United States

## Abstract

Elder care in rural Appalachia is challenging due to poor socioeconomic conditions, geographical isolation, and lack of services and transportation. Certain aspects of Appalachian culture, namely self-reliance, traditionalism, and strong family ties, also create unique barriers for using services to help care for persons with dementia (PwD). Quantitative and qualitative data from 85 caregivers of PWD with moderate to high care needs were explored to examine caregivers’ use of personal care services, identification with their community, attitudes towards service use, and geographical distance from the nearest service location. Results suggest that although services such as adult day centers, food banks, meal delivery, and support groups are widely dispersed throughout rural Appalachian counties, use or nonuse of services was driven by lack of economic resources, care preferences of the PwD, and dissatisfaction with previous service use. Discussion focuses on suggestions for uptake of services by caregivers of PwD in rural Appalachia.

